# Subclinical thyroid disorders should not be considered to be a non-classical risk factor for cardiovascular diseases

**DOI:** 10.1590/1516-3180.2020.0342.18112020

**Published:** 2021-01-08

**Authors:** Rodrigo Diaz Olmos

**Affiliations:** I MD, MSc, PhD. Assistant Professor, Department of Internal Medicine, School of Medicine, Universidade de São Paulo (USP), São Paulo (SP), Brazil.

Dear Editor,

I read with interest the editorial about subclinical thyroid disorders as a non-classical risk factor for cardiovascular disease.^1^ Although the editorial discusses some particularly important issues regarding the epidemiology of cardiovascular diseases and points out that there is a high burden of underdiagnosed subclinical thyroid diseases, especially among women and individuals with low socioeconomic status, some remarks about this need to be made.

There is no doubt about the importance of studying the association of subclinical thyroid disorders and cardiovascular risk. However, this association is weak and there is no evidence that treatment of these disorders is associated with reduced cardiovascular outcomes. Epidemiology, like science itself, is not value-free and it may be used as a tool to support predetermined ideas, as has been pointed out by many commentators.^2^ In the case of subclinical thyroid disorders, although many studies have shown that they have an association with surrogate markers for cardiovascular disease, their associations with clinical outcomes are less clear. The magnitude of the association is low and, hence, presence of such an association might only be a representation of residual confounding. Moreover, no randomized trial on treatment effect has been conducted.^3^

Sir Richard Doll has suggested that for an epidemiological study to be reasonably convincing, the lower limit of the 95% confidence level of increased risk should fall above a threefold increase (> 200% increase).^2^^,^^4^ Other authors have even suggested that a fourfold increase in risk should be the lower limit.^2^^,^^4^ On the other hand, subtler risks, such as the 30 to 50% increase in risk that has been observed in some studies on subclinical thyroid disorders, are not compelling. However, use of subclinical thyroid disorders as a novel cardiovascular risk factor would affect a large segment of the population (high prevalence of subclinical thyroid disorders) and have a potentially huge negative impact on public health, through transforming healthy people into sick people, without the expected benefits from treating a true risk factor.

We are increasingly embedded in a culture of overuse of medical services and medicalization of society. Our never-ending search for risk factors has been very favorable to and has been stimulated by the biomedical industry. Over the last few decades, we have gradually seen a change in preventive strategies such that high-risk strategies have become prioritized through reducing the cutoff points of traditional risk factors and creating new ones.

In fact, this sort of high-risk strategy has departed from the original strategy conceptualized by Geoffrey Rose almost three decades ago.^5^ This misinterpretation of Rose’s concept tries to encompass some of the characteristics of the original population strategy, to produce an intermediate type of prevention strategy that only exhibits the downsides of both the high-risk and the population strategy. It may also produce more fear and morbidity than what it is supposed to prevent, through transforming healthy people into sick individuals.

Therefore, I fear that attributing the status of a new cardiovascular risk to subclinical thyroid disorders is a misunderstanding that may reinforce the overmedicalization of an already medicalized society that perfectly suits a neoliberal approach to healthcare.^6^ This leads to labeling (i.e. heightened awareness of pseudo-risk factors), a range of psychological and physical side-effects, rising healthcare costs and overutilization of healthcare services without delivering better outcomes.

Healthcare needs to be directed towards sick and suffering people, such that prevention is left to interventions that are mostly outside the healthcare system. Indeed, the distinction between clinical care and prevention of future disease is essential for any robust healthcare system to thrive.
